# The measurement of splashover and carryover in centrifugal analyzers

**DOI:** 10.1155/S1463924679000559

**Published:** 1979

**Authors:** Peter Henry

**Affiliations:** Dept. of Medical Biochemistry Llandough Hospital South Glamorgan Wales Penarth CF6 1XX UK


### The measurement of splashover and carryover in centrifugal analyzers

**Peter Henry**

*Dept. of Medical Biochemistry, Llandough Hospital, Penarth, South Glamorgan, CF6 1XX, Wales.*

### Introduction

The principle of centrifugal fast analyzers was introduced by Anderson in a series of papers beginning in 1969 [1 - 4]. These analyzers were designed to handle several samples simultaneously and have proved to be very good at measuring rates of reaction. They use relatively small volumes of sample and reagent and hence they have become popular instruments for measuring enzyme activities.

Centrifugal analyzers have a multiple-cuvette assembly mounted on a rotor which is rotated through a stationary photometric system. At the beginning of an analysis, samples and reagents are delivered by a pipetting unit into wells in the central part of a transfer disc. The transfer disc is then positioned on the rotor, and when this is rotated, the samples and reagents are transferred together, by the centrifugal force generated, into the cuvettes which are arranged around the periphery. The contents of the cuvettes are then further mixed in some models by bubbles of air which are drawn through the cuvettes for a second or two.

Each cuvette passes in rapid sequence through the photometric system, and so the instrument combines the simplicity of a single beam system with the continuous referencing of a double beam system. A wealth of data is produced (about 10,000 data points per minute) and this is fed into a dedicated computer or calculator for the computation of the results.

Several models are commercially available, having from 15 to 36 cuvettes, and they can be classified into two types. In the first type, the cuvettes are part of the analyzer unit, and both transfer disc and cuvettes have to be laundered after each analysis. A typical block diagram is shown in Figure 1. In the second type the transfer disc and cuvettes are one integral and disposable unit.

The absorbance measured in any cuvette of a centrifugal analyzer may be affected by traces of material from both adjacent cuvettes. This contamination arises from two different effects, namely carryover and splashover. Carryover originates in the pipettor, and is due to traces of one sample being left in or around the pipettor tip and then being expelled with the following sample. Since it is not possible for material to be transferred from one cuvette to the preceding one, this process can be regarded as unidirectional.

The term splashover is used to describe an effect which is peculiar to the centrifugal analyzers which use bubble mixing. During this mixing, material can be transferred from a cuvette to either of its neighbours, and thus this process can be regarded as bidirectional.

The amount of material splashed over is governed by two main factors : the nature of the reagents and the suction force which is applied to create the mixing bubbles. A suction force which is quite satisfactory with some reagents may be quite unsuitable for use with reagents which easily froth. It is evident by the nature of the process that the splashover will vary considerably from one cuvette to the next. Consequently, a measurement of splashover for a given reagent and suction force would best be an average figure taken over all the cuvettes.

Measurements of carryover in centrifugal analyzers have been made (for example, by Henry *et al* [5]) using the method recommended by Young *et al* [6] for continuous flow analyzers. In this method, a series of six samples is repeated until all the positions in the ring are filled. The series consists of three samples ($h_1$, $h_2$ and $h_3$) of a serum specimen containing a high level of the substance under test followed by three samples ($l_1$, $l_2$ and $l_3$) of a serum specimen containing a low level of the substance. The carryover is measured as the percentage interaction, I, which for continuous flow analyzers is calculated from the mean of the two expressions:—

$$I_{\text{high to low}} = \frac{l_1 - l_3}{h_3 - l_3} \times 100\%$$

$$I_{\text{low to high}} = \frac{h_3 - h_1}{h_3 - l_3} \times 100\%$$

The same expressions are acceptable for centrifugal analyzers, but, on theoretical grounds, a denominator of ($h_2 - l_2$) would be preferable. Splashover was ignored in the earlier measurements [5], but fortunately, as will be shown in the theoretical section, the effect on the value of carryover calculated is small and can be ignored.

Although the occurrence of splashover is recognized by many users of centrifugal analyzers, there is no reference in the literature to its measurement. Sometimes an empirical check is made by running a series of dye solutions in the same pattern as for carryover. The transfer disc is filled manually so that there is no carryover and $h_1$ and $h_3$ are compared with $h_2$. If the two outer absorbance values are appreciably smaller than the middle one, then it is considered that the suction force is too great. The suction is then reduced until there is no apparent difference between $h_1$, $h_2$ and $h_3$.

This approach clearly will only be a rough guide because it uses aqueous dye solutions. It is not applicable to real situations involving reagents because the manual pipetting of 5 or 10 $\mu$l of serum is not sufficiently precise. The use of radioactive tracers is not feasible with most centrifugal analyzers that employ bubble mixing, because once the samples and reagents have begun to spin, there is no means of extracting the contents of individual cuvettes separately.

The amount of material displaced by splashover can, however, be calculated using the data already obtained in a determination of carryover. Since the first sample of each group of three is affected by carryover from the other group, whereas the 2nd and 3rd samples are only affected by carryover from samples with the same initial concentration, the splashover is calculated from the 2nd and 3rd samples. The Percentage Splashover is defined here to be 'that percentage of material which is splashed out from a cuvette and into one of its neighbours due to the forces taking place at the beginning of an analysis on a centrifugal analyzer'.

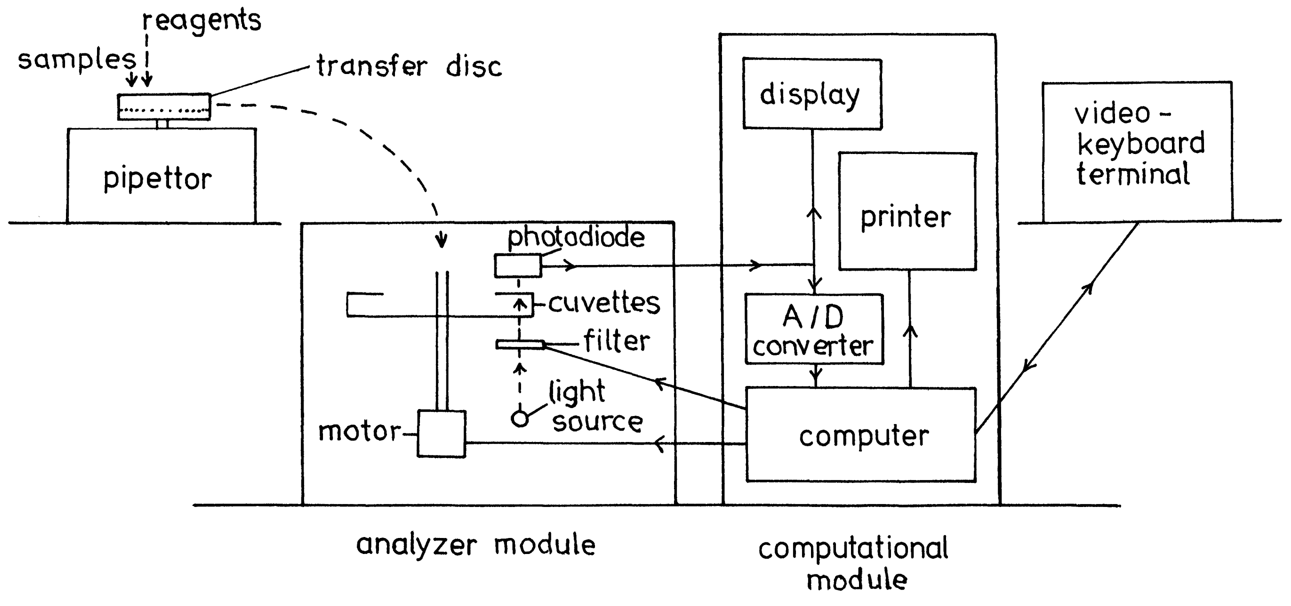

*Figure 1. Block diagram of a centrifugal analyzer.*

It can be calculated from the expressions:–

$$S_{\text{high to low}} = \frac{l_3 - l_2}{h_2 - l_2} \times 100\%$$

$$S_{\text{low to high}} = \frac{h_2 - h_3}{h_2 - l_2} \times 100\%$$

If these expressions give different numerical answers, then the Percentage Splashover is taken to be the mean of the two.

### Theory

Let the true concentrations of the specimens containing high and low amounts of substance be H and L, respectively, and the total volume of sample and reagents which would be in each cuvette if there were no carryover or splashover be V. Imagine a sequence of specimens : LHHHLLLHHHLLLHH-HLLLHHHLLLHHHLLLH in cuvettes 0, 1, 2 . . . . 31 giving results with concentrations $r_0$, $r_1$, $r_2$ . . . . $r_{31}$. Let the volumes carried over be $u_0, u_1, u_2$ . . . . $u_{31}$ and the volumes splashed over (in both directions) be $v_0$, $v_1$, $v_2$ . . . . $v_{31}$. Then, assuming that carryover and splashover only affect adjacent cuvettes as described, it follows that, for example

$$r_1 = \frac{HV + u_0L - u_1H + v_0L - 2v_1H + v_2H}{V + u_0 - u_1 + v_0 - 2v_1 + v_2}$$

Since the volume carried over from one compartment to the next is approximately constant, we can write $u = u_0 = u_1 = \ldots . u_{31}$ and therefore

$$r_1 = \frac{HV + uL - uH + v_0L - 2v_1H + v_2H}{V + v_0 - 2v_1 + v_2}$$

It is likely that some cuvettes splash out a greater volume than others. However, if the splashing is at random, the mean volume splashed out of every sixth cuvette starting with cuvette 1 will be approximately the same as the mean volumes splashed out of every sixth cuvette starting with cuvettes 2, 3, 4, 5 and 6 (cuvettes 0 and 31 being omitted in the calculations). That is,

$$v = 1/5\,(v_1 + v_7 + v_{13} + v_{19} + v_{25}) \doteqdot 1/5\,(v_2 + v_8 + v_{14} + v_{20} + v_{26}) \doteqdot \text{etc.}$$

Let the mean of $r_1$, $r_7$, $r_{13}$, $r_{19}$ and $r_{25}$ be $h_1$, the mean of $r_4$, $r_{10}$, $r_{16}$, $r_{22}$ and $r_{28}$ be $l_1$ etc., then it follows that

$$h_1 = \frac{HV\,\text{[illegible]}L - uH + vL - 2vH + vH}{V}$$

$$= H + \frac{1}{V}(uL - uH + vL - vH)$$

The expression may be further simplified by making the fraction carried over $(\frac{u}{V}) = c$ and the fraction splashed over $(\frac{v}{V}) = s$

then $h_1 = H + cL - cH + sL - sH$.

The following may be derived similarly:–

$h_2 = H$

$h_3 = H - sH + sL$

$l_1 = L + cH - cL + sH - sL$

$l_2 = L$

$l_3 = L + sH - sL$

It can be seen that

$l_1 - l_3 = h_3 - h_1 = c(H - L)$

$h_3 - l_3 = (1 - 2s)(H - L)$

$h_2 - l_2 = H - L$

$l_3 - l_2 = h_2 - h_3 = s(H - L)$

and hence that

$$\frac{l_1 - l_3}{h_3 - l_3} = \frac{h_3 - h_1}{h_3 - l_3} = \frac{c}{1 - 2s}$$

$$\frac{l_1 - l_3}{h_2 - l_2} = \frac{h_3 - h_1}{h_2 - l_2} = c$$

$$\frac{l_3 - l_2}{h_2 - l_2} = \frac{h_2 - h_3}{h_2 - l_2} = s$$

The precision of the measurements may be estimated by calculating values of c and s using the actual concentrations observed in each group of six specimens (three high and three low) rather than the mean values. Since splashover is assumed to occur at random, these calculations will be expected to produce some negative results. For example, if in one group of six, the only splashover was from cuvette $h_2$, the values calculated for $\frac{l_3 - l_2}{h_2 - l_2}$ and $\frac{h_2 - h_3}{l_2 - l_2}$

would be 0 and $-\frac{3sH}{H - L}$.

### Practical

During an evaluation of the Rotochem IIa(36), which is manufactured by the American Instrument Company, the need arose for an accurate assessment of splashover. The instrument had been adjusted by the engineer at the installation, using the empirical check method mentioned earlier, with dye solutions. However, splashover was suspected during routine running because of the values obtained for the four samples adjacent to two samples of patients' specimens which had very high gamma glutamyl transferase (GGT) activities. The method described was developed to meet this need.

Two serum specimens were used, one having a high GGT activity of about 320 IU/l and the other a lower one of about 70 IU/l. The samples of the specimens were arranged in the sample ring in the configuration described in the Theory section. Since Broughton *et al* [7] recommended taking the mean of ten results, and the present method gives the mean of only five results for a 36 cuvette rotor, the run was repeated. (Unfortunately there was insufficient sample in one cup so the results from the second run were the mean of four results). The mean activities found are given in Table 1 and the splashover and carryover calculated from these in Table 2.

Because the splashover was found to be so great, the whole experiment was repeated with the suction source disconnected so that there would be no bubble mixing, although there was still mixing caused by the design of the transfer disc and cuvettes. The results of this experiment are also given in Tables 1 and 2.

As a result of these findings, splashover was measured for all the other tests being used on the analyzer. Aspartate amino transferase (AST) was found to have a splashover of about 1.7% and so this, too, was subsequently run without the bubble mixing. For glucose, total protein and urea the splashover was negligible (less than 0.4%).

It was noticed that although the carryover calculated from the first run was negative and that of the second was positive, the mean value for the nine results was near to the value obtained in the runs performed without the suction. The precisions of the measurements were estimated as described in the Theory section. As a comparison, data was used from two runs which had been made to check the precision of the instrument for GGT's. All the sample cups contained a serum with a GGT activity of about 130 IU/l for the first of these runs and about 21 IU/l for the second. Two sets of results were made up from these by taking alternately three high level and three low level results. The carryover and splashover calculated give a measure of the deviations produced by the precision inherent in the method. The precision results are given in Table 3.

### Conclusions

The theoretical section showed that splashover and carryover can be calculated from one set of data. This is so because the former is a bidirectional effect and the latter is a unidirectional effect. The prediction has been borne out in practice.

**Table 1 Mean values found for the samples of high and low GGT activity**

| Run | gamma-glutamyl transferase (IU/l) | | | | | |
|---|---|---|---|---|---|---|
| | $h_1$ | $h_2$ | $h_3$ | $l_1$ | $l_2$ | $l_3$ |
| Normal 1 | 312.8 | 314.0 | 311.8 | 73.4 | 69.4 | 74.8 |
| Normal 2 | 318.75 | 324.75 | 321.25 | 80.25 | 73.25 | 78.25 |
| No suction 1 | 318.0 | 318.0 | 317.8 | 72.2 | 71.6 | 72.0 |
| No suction 2 | 317.8 | 318.2 | 317.4 | 72.2 | 72.0 | 71.6 |

**Table 2 Carryover and splashover values for GGT**

| Run | Carryover % | | | | | | Splashover % | | |
|---|---|---|---|---|---|---|---|---|---|
| | using $(h_3 - l_3)$ | | | using $(h_2 - l_2)$ | | | | | |
| | h → l | l → h | Mean | h → l | l → h | Mean | h → l | l → h | Mean |
| Normal 1 | −.59 | −.42 | .13 | −.57 | −.41 | .13 | 2.21 | 0.90 | 1.61 |
| Normal 2 | .82 | 1.03 | | .80 | .99 | | 1.99 | 1.19 | |
| No suction 1 | .08 | −.08 | .02 | .08 | −.08 | .02 | .16 | .08 | .10 |
| No suction 2 | .24 | −.16 | | .24 | −.16 | | −.16 | .32 | |

**Table 3 Precisions of carryover and splashover**

| Type of Run | No. of estimates | Standard Error of the Mean | | 95% confidence limits for the Mean | |
|---|---|---|---|---|---|
| | | Carryover | Splashover | Carryover | Splashover |
| Normal | 18 | 0.52 | 0.46 | −0.9 to +1.2 | +0.7 to +2.5 |
| No Suction | 20 | 0.07 | 0.09 | −0.1 to +0.2 | −0.1 to +0.3 |
| Made up | 20 | 0.17 | 0.19 | −0.4 to +0.3 | −0.3 to +0.4 |

Splashovers of the order of 2% were measured for GGT and AST when the analyzer was run normally, but these splashovers were reduced to about 0.1% by running the analyzer without the bubble mixing. The mixing obtained without the bubbles is still quite adequate for these enzymes. With GGT, for example, the coefficient of variation, within batch, was 0.9% at a level of 57 IU/l.

Since splashover is an irregular phenomenon which will affect one cuvette more than another, negative values for splashover are not infrequently obtained. Another reason for the negative values is that the amount of splashover encountered is 2% or less, which is of the same order as the precision of the measurements. The "made up" runs in Table 3 show that mean values of less than 0.4% are not significantly different from zero.

Table 3 also shows that, as would be expected, the carryover values calculated in a run with a low splashover are much more precise than those calculated in a run with a high splashover. The standard error of the mean was at least seven times smaller when the GGT was run without the bubble mixing. However, if enough repeats are made, the mean values for carryover will be the same whether there is high or low splashover.

The percentage interaction figures given in Table 2 show that either $(h_2 - l_2)$ or $(h_3 - l_3)$ may be used as the denominator in calculating the carryover. For centrifugal analyzers, where splashover is being calculated as well as carryover, the use of $(h_2 - l_2)$ is both easier and more theoretically satisfying.

The splashover should be measured for every test done on a centrifugal analyzer, since the amount of splashover depends upon the reagent as well as the suction force. No appreciable splashover should be tolerated because if the blank, standard or reference cuvettes are contaminated, all the results will be wrong.

ACKNOWLEDGEMENTS
The author is grateful to Mr. W. A. White for technical assistance, to V. A. Howe & Co. Ltd., for the loan of the instrument and to the Department of Health and Social Security for their permission to publish this article.

REFERENCES
[1] Anderson, N.G., (1969) *Analytical Biochemistry,* **28,** 545.
[2] Anderson, N.G., (1969) *Analytical Biochemistry,* **32,** 59.
[3] Anderson, N.G., (1969) *Science,* **166,** 317.
[4] Anderson, N.G., (1970) *American Journal of Clinical Pathology* **52,** 778.
[5] Henry, P. and Saunders, R.A., (1976) *Annals of Clinical Biochemistry,* **13,** 384.
[6] Young, D.S. and Gochman, N., in "Standard Methods of Clinical Chemistry" volume 7 Ed. Cooper G.R. 1972 Academic Press, p. 303.
[7] Broughton, P.M.G., Buttolph, M.A., Gowenlock, A.H., Neill, D.W. and Skentelbery, R.G., (1969) *Journal of Clinical Pathology* **22,** 278.

### A microprocessor controlled liquid chromatograph/atomic absorption sampling system

**Thomas M. Vickrey and William Eue.**

*Department of Chemistry, Texas A & M University, College Station, Texas 77843, USA.*

### Introduction
The need for molecular characterization of metal-containing species at the trace level has led to the interfacing of high resolution chromatographic techniques with element-specific detectors [1, 2, 3]. One such system is the interfacing of liquid chromatography (LC) to atomic absorption spectroscopy (AA). The resultant instrumentation (LCAA) is capable of specific characterization (speciation) of the metal-containing compounds and detection of the separate species at the nanogram level [3]. The application of LCAA with a graphite furnace atomic absorption spectrometer has been reported [1, 2]. The use of a graphite furnace atomic absorption spectrometer as a detector for a liquid chromatograph has technical problems associated with it. However, the ability to monitor biological transport of metal-containing species in the environment, speciate clinically important metal compounds, or to use metal complexation as a tag for nonmetal species which are otherwise difficult to detect [4], make this technique attractive.

This work presents a versatile controller for the sampling interface, using a 6800 based microprocessor system. This interface controller is software programable to operate either in the 'pulsed' mode, or in the 'total consumption' mode. The hardware and software descriptions are presented, as well as the results of sampling precision studies.

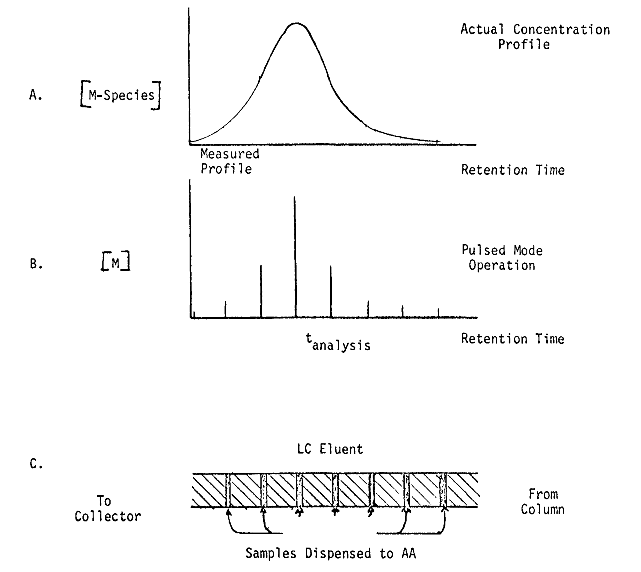

*Figure 1. Description of the "pulsed" sampling mode. A, actual concentration profile of metallospecies leaving the column. B, the measured concentration profile from the intermittent removal of aliquots of the eluent stream. C, a diagram of the eluent stream showing the aliquots which are removed and analyzed by the graphite furnace atomic absorption spectrometer.*

### Three sampling modes
There are three basic modes for LCAA operation: survey, pulsed and total consumption. In the survey mode the sample is chromatographed, fractions collected and the collected fractions analyzed for the metal of interest. This is the simplest form of the LCAA experiment and requires only the 'human' interface. The pulsed mode operates during the chromatographic run; the eluent stream is periodically sampled and the sample dispensed into a graphite furnace for the AA analysis cycle as shown in Figure 1. The term 'pulsed'

